# Dendritic cell targeting vaccine for HPV-associated cancer

**Published:** 2017-01-15

**Authors:** Wenjie Yin, Dorothée Duluc, HyeMee Joo, SangKon Oh

**Affiliations:** 1 Baylor Institute for Immunology Research, 3434 Live Oak Street, Dallas, TX 75204, USA; 2 Institute of Biomedical Studies, Baylor University, South 5th Street, Waco, TX 76706, USA

**Keywords:** Dendritic cell, CD40, Cross-presentation, Lectin, Vaccine, Cancer, Immunotherapy, HPV

## Abstract

Dendritic cells (DCs) are major antigen presenting cells that can efficiently prime and activate cellular immune responses. Delivering antigens to *in vivo* DCs has thus been considered as a promising strategy that could allow us to mount T cell-mediated therapeutic immunity against cancers in patients. Successful development of such types of cancer vaccines that can target *in vivo* DCs, however, requires a series of outstanding questions that need to be addressed. These include the proper selection of which DC surface receptors, specific DC subsets and DC activators that can further enhance the efficacy of vaccines by promoting effector T cell infiltration and retention in tumors and their actions against tumors. Supplementing these areas of research with additional strategies that can counteract tumor immune evasion mechanisms is also expected to enhance the efficacy of such therapeutic vaccines against cancers. After more than a decade of study, we have concluded that antigen targeting to DCs via CD40 to evoke cellular responses is more efficient than targeting antigens to the same types of DCs via eleven other DC surface receptors tested. In recent work, we have further demonstrated that a prototype vaccine (anti-CD40-HPV16.E6/7, a recombinant fusion protein of anti-human CD40 and HPV16.E6/7 protein) for HPV16-associated cancers can efficiently activate HPV16.E6/7-specific T cells, particularly CD8^+^ T cells, from the blood of HPV16^+^ head-and-neck cancer patients. Moreover, anti-CD40-HPV16.E6/7 plus poly(I:C) can mount potent therapeutic immunity against TC-1 tumor expressing HPV16.E6/7 protein in human CD40 transgenic mice. In this manuscript, we thus highlight our recent findings for the development of novel CD40 targeting immunotherapeutic vaccines for HPV16-associated malignancies. In addition, we further discuss several of key questions that still remain to be addressed for enhancing therapeutic immunity elicited by our prototype vaccine against HPV16-associated malignancies.

Dendritic cells (DCs) are the major antigen presenting cells (APCs) that can efficiently cross-prime antigen-specific CD8^+^ T cells ^[1, 2]^. Such functional specialty in turn makes DCs the ideal cellular targets for the rational design of vaccine against cancers. In line with these notions, Bonifaz *et al.* demonstrated that antigen targeting to *in vivo* DCs via DEC-205 using conjugates of anti-DEC-205 and antigen is far more efficient than antigen alone at eliciting antigen-specific cellular immunity ^[3]^.

For more than a decade after the initial report on DC targeting vaccines ^[3]^, groups of scientists have been trying to optimize DC-targeting vaccines by delivering antigens to different DC surface receptors. These receptors include c-type lectins (e.g., DEC205, DC-SIGN, CD207, LOX-1, DC-ASGPR, Dectin-1, DCIR, DCIR2, CLEC6, CLEC9A, and CLEC12A) ^[3–22]^, as well as non-lectin receptors, including CD40 ^[22–26]^, mannose receptor ^[27]^, and integrins ^[28]^. Antigens delivered to DCs via these receptors have been shown to elicit certain levels of antigen-specific CD8^+^ CTL responses *in vitro* in humans and/or *in vivo* in mice or non-human primates (NHPs). However, it remains unclear which targeted receptors are the most efficient at priming and boosting antigen-specific CD8^+^ and CD4^+^ T cell responses. Finding a specific DC surface receptor through which potent T cell responses, particularly CD8^+^ T cell responses, can be elicited is fundamental for the rational design and development of effective DC-targeting vaccines against cancers.

In our previous study ^[29]^, we tested 11 different human DC surface receptors (CD40, LOX-1, Dectin-1, DEC-205, DC-ASGPR, DC-SIGN, DC-SIGN/L, DCIR, CLEC6, MARCO, and CD1d) for their ability to elicit antigen-specific CD8^+^ T cell responses. We found that CD40 was the most efficient at both priming and boosting antigen-specific functional CD8^+^T cell responses in a human *in vitro* system. Interestingly, however, lectin-like receptors (LOX-1 and Dectin-1) were more efficient than CD40 at eliciting antigen-specific CD4^+^ T cell responses in a human *in vitro* system. *In vivo* data generated in mice also showed that CD40 was more efficient than Langerin at eliciting antigen-specific CD8^+^ T cell responses; whereas Langerin, another lectin-like receptor, was more efficient than CD40 at eliciting antigen-specific CD4^+^ T cell responses. Although antigens fused to anti-CD40 and anti-Langerin antibodies may not target the same subsets of DCs in mice, these data further support our conclusion that antigen targeting to DCs via CD40 is an efficient way to elicit antigen-specific CD8^+^ T cell responses.

We further investigated the functional differences between CD40 and lectins in antigen presentation to CD8^+^ and CD4^+^ T cells by examining the subcellular and intracellular trafficking of the three different receptor-bound mAbs in DCs. Anti-CD40 mAb was present mainly on the cell membrane and in early endosomal compartments, which likely contributed to the enhanced antigen cross-presentation to CD8^+^ T cells ^[23, 24]^. On the other hand, anti-LOX-1 and anti-Dectin-1 localized to both the early and late endosomal compartments. These late endosomal compartments are less efficient for antigen cross-presentation due to a higher concentration of lysosomal enzymes that degrade antigens before they can escape into the cytosol. They are still able to contribute to antigen cross-presentation, especially when their proteolysis is inhibited ^[24]^. We also found that a large fraction of anti-CD40 mAb remained at the plasma membrane, whereas the majority of both anti-LOX-1 and anti-Dectin-1 mAbs were rapidly internalized into endosomal vesicles. Slow internalization to early endosomes or rapid antigen recycling, as described previously ^[23, 24]^, could result in increased antigen stability, followed by prolonged antigen presentation and enhanced CD8^+^ T cell responses. In line with this, we found that CD40 targeting leads to greater as well as prolonged antigen cross-presentation to CD8^+^ T cells compared to LOX-1 or Dectin-1 targeting.

To move forward our efforts for the development of CD40-targeting vaccines against cancer, we generated multiple recombinant proteins of humanized anti-CD40 antibody carrying different tumor-associated antigens (TAAs), including prostate-specific antigens and HPV16.E6/7 protein, as prototype vaccines. In our recent studies ^[29, 30]^, we have further demonstrated the proof of concept that strongly support the clinical development of CD40-targeting therapeutic vaccines for HPV16-associated cancers using our prototype vaccine, anti-CD40-HPV16.E6/7 protein.

Studies have shown that 79 million people are infected with HPV, with 14 million new cases of infections each year in the United States ^[31, 32]^. Of the more than 150 different types of HPV ^[33]^, high-risk HPV strains (HPV16 and 18) are strongly associated with many cancers of the cervix, vagina, vulva, penis, and anus ^[34, 35]^. Up to 22% of adults are HPV16-seropositive, but most primary infections are cleared without sequelae ^[36–38]^. However, in a small but significant proportion of individuals, their immune systems fail to eradicate the virus and it becomes latent. Such persistent infection can lead to cancers. A recent U.S. population-based study conducted by the Centers for Disease Control and Prevention shows that 66% of cervical cancers, 55% of vaginal cancers, 79% of anal cancers, and 62% of oropharyngeal cancers are attributable to HPV16 or 18. Among these cancers, HPV-associated head-and-neck cancers, including oropharyngeal squamous cell carcinomas, have recently risen dramatically in men under 50 years old; whereas the incidence of HPV-negative oropharyngeal cancers has decreased ^[39]^. Although many of the HPV-positive tumors can be cured with modern multidisciplinary treatment approaches, development of new and effective therapeutic vaccines against HPV-associated malignancies is of importance to bring better clinical benefit to cancer patients. Currently available prophylactic vaccines with viral capsid proteins are not effective for the treatment of HPV-associated cancer ^[40]^.

Malignant transformation of HPV-infected cells is driven by the products of HPV oncoproteins *E6* and *E7* (E6/7), which inactivate p53 and the retinoblastoma tumor suppressor genes ^[41]^. All HPV-infected cells constitutively express TAAs of viral origin, such as HPV E6/7. As a result, E6/7 are fitting target TAAs for evoking HPV-specific cellular immune responses, particularly CD8^+^ cytotoxic T lymphocytes (CTLs), which are one of the major tumoricidal effector cell types. Multiple candidate vaccines targeting E6/7 are currently under development, including E6- and/or E7-derived peptide ^[42–45]^, protein ^[46, 47]^, plasmid ^[48, 49]^ and live-vectored vaccines ^[50–53]^. Nevertheless, potential safety concerns for some of these vaccine models, especially for immunocompromised individuals, and the overall weak CD8^+^ CTL-mediated immunity-remain major challenges in developing a safe and effective vaccine against HPV-associated malignancies ^[54–56]^.

Critical findings in our recent study ^[30]^ with a prototype vaccine for HPV16-associated malignancies, anti-CD40-HPV16.E6/7, include that it can evoke HPV16.E6/7-specific CD8^+^ and CD4^+^ T cell responses in head-and-neck cancer patients *in vitro* and in human CD40 transgenic (hCD40tg) mice *in vivo*. The combination of anti-CD40-HPV16.E6/7 and poly(I:C) efficiently primed HPV16.E6/7-specific T cells, particularly CD8^+^ T cells, in hCD40tg mice and could thus mount therapeutic immunity against challenged tumors. The observed therapeutic immunity evoked with the prototype vaccine was associated with the frequency of HPV16.E6/7-specific CD8^+^ T cells in the tumors but not in the blood. Taken together, these data suggest that CD40-targeting vaccines for HPV-associated malignancies can provide a highly immunogenic platform with a strong likelihood of clinical benefit. Data from this study offer strong support for the development of CD40-targeting vaccines for other cancers in the future.

To achieve our ultimate goals with this prototype therapeutic vaccine for HPV16-associated malignancies, however, we still need to consider several factors that could also determine the success or failure of clinical development of this prototype vaccine. These factors include 1) selection of proper adjuvant, 2) route of immunization, 3) finding strategies to promote effector CD8^+^ T cell migration into mucosal tissues, and 4) finding strategies to efficiently counteract tumor immune evasion mechanisms. Throughout our studies ^[30]^, we have used poly(I:C) as an adjuvant, and this decision was based on our human *in vitro* data ^[30]^ and mouse *in vivo* data from previous studies ^[57, 58]^. Recent studies in NHPs and humans have also shown that poly(I:C) and it’s derivative poly-ICLC are safe ^[59]^ and can thus be used as an adjuvant for our prototype vaccine for HPV16-associated cancers. In our study ^[30]^, we also found that poly(I:C) in montanide resulted in significantly enhanced HPV16-E6/7-specific CD4^+^ T cell responses, although there was no significant change in the magnitude of CD8^+^ T cell responses. If this is the case in humans, montanide might improve the efficacy of this vaccine model in patients because of the critical roles of CD4^+^ T cells in the maintenance of CD8^+^ CTLs ^[60–62]^. Other DC activators, including toll-like receptor (TLR) 7/8 ligands, particularly in the form of conjugates to the prototype vaccine, might also promote CD8^+^ T cell-mediated immunity, as described ^[63, 64]^.

Also, the route of immunization could be important in patients, although s.c., i.p., and i.m. delivery of anti-CD40-HPV16.E6/7 plus poly(I:C) resulted in similar outcomes in mice. Alternatively, an experimental protocol using NHPs may provide us with better insights for the selection of an optimal immunization route for evoking strong CD8^+^ T cell responses.

It will also be important to test whether this vaccine model is capable of evoking mucosal CD8^+^ CTL-mediated immunity. This might be one of the most important and relevant tasks for the successful development of vaccines against HPV-associated cancers in the mucosa. This question may need to be addressed in the context of selecting the optimal immunization route as well as choosing appropriate adjuvants.

Lastly, the efficacy of this vaccine model might be improved by harnessing tumor immune evasion mechanisms. Although HPV-induced tumors in different mucosal tissues might possess distinct inhibitory mechanisms in patients (and these need to be further studied), recent data suggest that antibodies specific for immune checkpoint inhibitors, including CTLA-4, PD-1, and PD-L1 ^[65–68]^, might also improve the efficacy of this vaccine in patients with HPV-associated malignancies.

In summary, data from our recent studies ^[29, 30]^ strongly support the continued clinical development of a CD40-targeting therapeutic vaccine for HPV16-associated malignancies. However, it is also important to note that the chance of successful development of this vaccine will also be largely dependent on multiple factors that have been discussed in this manuscript. Nonetheless, data from our and other recent studies ^[22–24, 29]^ support the development of CD40-targeting vaccines against other cancers.

## Abbreviations

HPV: human papillomavirus
TAA: tumor-associated antigen
CTL: cytotoxic T lymphocyte
DC: dendritic cell
APC: antigen-presenting cell
NHP: non-human primate
mAb: monoclonal antibody
MHC: major histocompatibility complex
Poly(I:C): polyinosinic:polycytidylic acid
PBMC: peripheral blood mononuclear cell
hCD40tg: human CD40 transgenic
TLR: toll-like receptor

## References

[1] Delamarre L, Mellman I (2011). Harnessing dendritic cells for immunotherapy. Semin Immunol.

[2] Jung S, Unutmaz D, Wong P, Sano G, De los Santos K, Sparwasser T (2002). In vivo depletion of CD11c+ dendritic cells abrogates priming of CD8+ T cells by exogenous cell-associated antigens. Immunity.

[3] Bonifaz LC, Bonnyay DP, Charalambous A, Darguste DI, Fujii S, Soares H (2004). In vivo targeting of antigens to maturing dendritic cells via the DEC-205 receptor improves T cell vaccination. J Exp Med.

[4] Sancho D, Mourao-Sa D, Joffre OP, Schulz O, Rogers NC, Pennington DJ (2008). Tumor therapy in mice via antigen targeting to a novel, DC-restricted C-type lectin. J Clin Invest.

[5] Caminschi I, Proietto AI, Ahmet F, Kitsoulis S, Shin Teh J, Lo JC (2008). The dendritic cell subtype-restricted C-type lectin Clec9A is a target for vaccine enhancement. Blood.

[6] Idoyaga J, Cheong C, Suda K, Suda N, Kim JY, Lee H (2008). Cutting edge: langerin/CD207 receptor on dendritic cells mediates efficient antigen presentation on MHC I and II products in vivo. J Immunol.

[7] Delneste Y, Magistrelli G, Gauchat J, Haeuw J, Aubry J, Nakamura K (2002). Involvement of LOX-1 in dendritic cell-mediated antigen cross-presentation. Immunity.

[8] Li D, Romain G, Flamar AL, Duluc D, Dullaers M, Li XH (2012). Targeting self- and foreign antigens to dendritic cells via DC-ASGPR generates IL-10-producing suppressive CD4+ T cells. J Exp Med.

[9] Weck MM, Appel S, Werth D, Sinzger C, Bringmann A, Grunebach F (2008). hDectin-1 is involved in uptake and cross-presentation of cellular antigens. Blood.

[10] Carter RW, Thompson C, Reid DM, Wong SY, Tough DF (2006). Preferential induction of CD4+ T cell responses through in vivo targeting of antigen to dendritic cell-associated C-type lectin-1. J Immunol.

[11] Ni L, Gayet I, Zurawski S, Duluc D, Flamar AL, Li XH (2010). Concomitant activation and antigen uptake via human dectin-1 results in potent antigen-specific CD8+ T cell responses. J Immunol.

[12] Tacken PJ, de Vries IJ, Gijzen K, Joosten B, Wu D, Rother RP (2005). Effective induction of naive and recall T-cell responses by targeting antigen to human dendritic cells via a humanized anti-DC-SIGN antibody. Blood.

[13] Dudziak D, Kamphorst AO, Heidkamp GF, Buchholz VR, Trumpfheller C, Yamazaki S (2007). Differential antigen processing by dendritic cell subsets in vivo. Science.

[14] Idoyaga J, Lubkin A, Fiorese C, Lahoud MH, Caminschi I, Huang Y (2011). Comparable T helper 1 (Th1) and CD8 T-cell immunity by targeting HIV gag p24 to CD8 dendritic cells within antibodies to Langerin, DEC205, and Clec9A. Proc Natl Acad Sci U S A.

[15] Tacken PJ, Ginter W, Berod L, Cruz LJ, Joosten B, Sparwasser T (2011). Targeting DC-SIGN via its neck region leads to prolonged antigen residence in early endosomes, delayed lysosomal degradation, and cross-presentation. Blood.

[16] Meyer-Wentrup F, Benitez-Ribas D, Tacken PJ, Punt CJ, Figdor CG, de Vries IJ (2008). Targeting DCIR on human plasmacytoid dendritic cells results in antigen presentation and inhibits IFN-alpha production. Blood.

[17] Tacken PJ, de Vries IJ, Torensma R, Figdor CG (2007). Dendritic-cell immunotherapy: from ex vivo loading to in vivo targeting. Nat Rev Immunol.

[18] Kastenmuller W, Kastenmuller K, Kurts C, Seder RA (2014). Dendritic cell-targeted vaccines--hope or hype?. Nat Rev Immunol.

[19] Lahoud MH, Proietto AI, Ahmet F, Kitsoulis S, Eidsmo L, Wu L (2009). The C-type lectin Clec12A present on mouse and human dendritic cells can serve as a target for antigen delivery and enhancement of antibody responses. J Immunol.

[20] Flacher V, Tripp CH, Mairhofer DG, Steinman RM, Stoitzner P, Idoyaga J (2014). Murine Langerin+ dermal dendritic cells prime CD8+ T cells while Langerhans cells induce cross-tolerance. EMBO Mol Med.

[21] Duluc D, Joo H, Ni L, Yin W, Upchurch K, Li D (2014). Induction and activation of human Th17 by targeting antigens to dendritic cells via dectin-1. J Immunol.

[22] Flamar AL, Xue Y, Zurawski SM, Montes M, King B, Sloan L (2013). Targeting concatenated HIV antigens to human CD40 expands a broad repertoire of multifunctional CD4+ and CD8+ T cells. Aids.

[23] Cohn L, Chatterjee B, Esselborn F, Smed-Sorensen A, Nakamura N, Chalouni C (2013). Antigen delivery to early endosomes eliminates the superiority of human blood BDCA3+ dendritic cells at cross presentation. J Exp Med.

[24] Chatterjee B, Smed-Sorensen A, Cohn L, Chalouni C, Vandlen R, Lee BC (2012). Internalization and endosomal degradation of receptor-bound antigens regulate the efficiency of cross presentation by human dendritic cells. Blood.

[25] Rosalia RA, Cruz LJ, van Duikeren S, Tromp AT, Silva AL, Jiskoot W (2015). CD40-targeted dendritic cell delivery of PLGA-nanoparticle vaccines induce potent anti-tumor responses. Biomaterials.

[26] Williams BJ, Bhatia S, Adams LK, Boling S, Carroll JL, Li XL (2012). Dendritic cell based PSMA immunotherapy for prostate cancer using a CD40-targeted adenovirus vector. PLoS One.

[27] Tsuji T, Matsuzaki J, Kelly MP, Ramakrishna V, Vitale L, He LZ (2011). Antibody-targeted NY-ESO-1 to mannose receptor or DEC-205 in vitro elicits dual human CD8+ and CD4+ T cell responses with broad antigen specificity. J Immunol.

[28] Castro FV, Tutt AL, White AL, Teeling JL, James S, French RR (2008). CD11c provides an effective immunotarget for the generation of both CD4 and CD8 T cell responses. Eur J Immunol.

[29] Yin W, Gorvel L, Zurawski S, Li D, Ni L, Duluc D (2016). Functional Specialty of CD40 and Dendritic Cell Surface Lectins for Exogenous Antigen Presentation to CD8(+) and CD4(+) T Cells. EBioMedicine.

[30] Yin W, Duluc D, Joo H, Xue Y, Gu C, Wang Z (2016). Therapeutic HPV Cancer Vaccine Targeted to CD40 Elicits Effective CD8+ T-cell Immunity. Cancer Immunol Res.

[31] Satterwhite CL, Torrone E, Meites E, Dunne EF, Mahajan R, Ocfemia MC (2013). Sexually transmitted infections among US women and men: prevalence and incidence estimates, 2008. Sex Transm Dis.

[32] Dunne EF, Markowitz LE, Saraiya M, Stokley S, Middleman A, Unger ER (2014). CDC Grand Rounds: Reducing the Burden of HPV-Associated Cancer and Diseases. MMWR.

[33] Bernard HU (2005). The clinical importance of the nomenclature, evolution and taxonomy of human papillomaviruses. J Clin Virol.

[34] D’Souza G, Kreimer AR, Viscidi R, Pawlita M, Fakhry C, Koch WM (2007). Case-control study of human papillomavirus and oropharyngeal cancer. N Engl J Med.

[35] Kreimer AR, Clifford GM, Boyle P, Franceschi S (2005). Human papillomavirus types in head and neck squamous cell carcinomas worldwide: a systematic review. Cancer Epidemiol Biomarkers Prev.

[36] de Sanjose S, Diaz M, Castellsague X, Clifford G, Bruni L, Munoz N (2007). Worldwide prevalence and genotype distribution of cervical human papillomavirus DNA in women with normal cytology: a meta-analysis. Lancet Infect Dis.

[37] Dunne EF, Nielson CM, Stone KM, Markowitz LE, Giuliano AR (2006). Prevalence of HPV infection among men: A systematic review of the literature. J Infect Dis.

[38] Woodman CB, Collins S, Winter H, Bailey A, Ellis J, Prior P (2001). Natural history of cervical human papillomavirus infection in young women: a longitudinal cohort study. Lancet.

[39] Sathish N, Wang X, Yuan Y (2014). Human Papillomavirus (HPV)-associated Oral Cancers and Treatment Strategies. J Dent Res.

[40] Roden R, Wu TC (2006). How will HPV vaccines affect cervical cancer?. Nat Rev Cancer.

[41] Munger K, Phelps WC, Bubb V, Howley PM, Schlegel R (1989). The E6 and E7 genes of the human papillomavirus type 16 together are necessary and sufficient for transformation of primary human keratinocytes. J Virol.

[42] Steller MA, Gurski KJ, Murakami M, Daniel RW, Shah KV, Celis E (1998). Cell-mediated immunological responses in cervical and vaginal cancer patients immunized with a lipidated epitope of human papillomavirus type 16 E7. Clin Cancer Res.

[43] de Vos van Steenwijk PJ, Ramwadhdoebe TH, Lowik MJ, van der Minne CE, Berends-van der Meer DM, Fathers LM (2012). A placebo-controlled randomized HPV16 synthetic long-peptide vaccination study in women with high-grade cervical squamous intraepithelial lesions. Cancer Immunol Immunother.

[44] Kenter GG, Welters MJ, Valentijn AR, Lowik MJ, Berends-van der Meer DM, Vloon AP (2009). Vaccination against HPV-16 oncoproteins for vulvar intraepithelial neoplasia. N Engl J Med.

[45] Voskens CJ, Sewell D, Hertzano R, DeSanto J, Rollins S, Lee M (2012). Induction of MAGE-A3 and HPV-16 immunity by Trojan vaccines in patients with head and neck carcinoma. Head Neck.

[46] Fausch SC, Da Silva DM, Eiben GL, Le Poole IC, Kast WM (2003). HPV protein/peptide vaccines: from animal models to clinical trials. Front Biosci.

[47] Gerard CM, Baudson N, Kraemer K, Bruck C, Garcon N, Paterson Y (2001). Therapeutic potential of protein and adjuvant vaccinations on tumour growth. Vaccine.

[48] Lin K, Roosinovich E, Ma B, Hung CF, Wu TC (2010). Therapeutic HPV DNA vaccines. Immunol Res.

[49] Peng S, Lyford-Pike S, Akpeng B, Wu A, Hung CF, Hannaman D (2013). Low-dose cyclophosphamide administered as daily or single dose enhances the antitumor effects of a therapeutic HPV vaccine. Cancer Immunol Immunother.

[50] Lin CW, Lee JY, Tsao YP, Shen CP, Lai HC, Chen SL (2002). Oral vaccination with recombinant Listeria monocytogenes expressing human papillomavirus type 16 E7 can cause tumor growth in mice to regress. Int J Cancer.

[51] Gunn GR, Zubair A, Peters C, Pan ZK, Wu TC, Paterson Y (2001). Two Listeria monocytogenes vaccine vectors that express different molecular forms of human papilloma virus-16 (HPV-16) E7 induce qualitatively different T cell immunity that correlates with their ability to induce regression of established tumors immortalized by HPV-16. J Immunol.

[52] Jabbar IA, Fernando GJ, Saunders N, Aldovini A, Young R, Malcolm K (2000). Immune responses induced by BCG recombinant for human papillomavirus L1 and E7 proteins. Vaccine.

[53] Maciag PC, Radulovic S, Rothman J (2009). The first clinical use of a live-attenuated Listeria monocytogenes vaccine: a Phase I safety study of Lm-LLO-E7 in patients with advanced carcinoma of the cervix. Vaccine.

[54] Kenter GG, Welters MJ, Valentijn AR, Lowik MJ, Berends-van der Meer DM, Vloon AP (2008). Phase I immunotherapeutic trial with long peptides spanning the E6 and E7 sequences of high-risk human papillomavirus 16 in end-stage cervical cancer patients shows low toxicity and robust immunogenicity. Clin Cancer Res.

[55] Stanley M (2008). Immunobiology of HPV and HPV vaccines. Gynecol Oncol.

[56] Welters MJ, Kenter GG, Piersma SJ, Vloon AP, Lowik MJ, Berends-van der Meer DM (2008). Induction of tumor-specific CD4+ and CD8+ T-cell immunity in cervical cancer patients by a human papillomavirus type 16 E6 and E7 long peptides vaccine. Clin Cancer Res.

[57] Bonifaz L, Bonnyay D, Mahnke K, Rivera M, Nussenzweig MC, Steinman RM (2002). Efficient targeting of protein antigen to the dendritic cell receptor DEC-205 in the steady state leads to antigen presentation on major histocompatibility complex class I products and peripheral CD8+ T cell tolerance. J Exp Med.

[58] Gurer C, Strowig T, Brilot F, Pack M, Trumpfheller C, Arrey F (2008). Targeting the nuclear antigen 1 of Epstein-Barr virus to the human endocytic receptor DEC-205 stimulates protective T-cell responses. Blood.

[59] Ammi R, De Waele J, Willemen Y, Van Brussel I, Schrijvers DM, Lion E (2015). Poly(I:C) as cancer vaccine adjuvant: Knocking on the door of medical breakthroughs. Pharmacology & Therapeutics.

[60] Sun JC, Bevan MJ (2003). Defective CD8 T cell memory following acute infection without CD4 T cell help. Science.

[61] Shedlock DJ, Shen H (2003). Requirement for CD4 T cell help in generating functional CD8 T cell memory. Science.

[62] Oh S, Perera LP, Terabe M, Ni L, Waldmann TA, Berzofsky JA (2008). IL-15 as a mediator of CD4+ help for CD8+ T cell longevity and avoidance of TRAIL-mediated apoptosis. Proc Natl Acad Sci U S A.

[63] Kastenmuller K, Wille-Reece U, Lindsay RW, Trager LR, Darrah PA, Flynn BJ (2011). Protective T cell immunity in mice following protein-TLR7/8 agonist-conjugate immunization requires aggregation, type I IFN, and multiple DC subsets. J Clin Invest.

[64] Wille-Reece U, Flynn BJ, Lore K, Koup RA, Kedl RM, Mattapallil JJ (2005). HIV Gag protein conjugated to a Toll-like receptor 7/8 agonist improves the magnitude and quality of Th1 and CD8+ T cell responses in nonhuman primates. Proc Natl Acad Sci U S A.

[65] Brahmer JR, Tykodi SS, Chow LQ, Hwu WJ, Topalian SL, Hwu P (2012). Safety and activity of anti-PD-L1 antibody in patients with advanced cancer. N Engl J Med.

[66] Topalian SL, Hodi FS, Brahmer JR, Gettinger SN, Smith DC, McDermott DF (2012). Safety, activity, and immune correlates of anti-PD-1 antibody in cancer. N Engl J Med.

[67] Honda T, Egen JG, Lammermann T, Kastenmuller W, Torabi-Parizi P, Germain RN (2014). Tuning of antigen sensitivity by T cell receptor-dependent negative feedback controls T cell effector function in inflamed tissues. Immunity.

[68] Pardoll DM (2012). The blockade of immune checkpoints in cancer immunotherapy. Nat Rev Cancer.

